# Supplementary material for: Transcriptome profiling uncovers differentially expressed genes linked to nutritional quality in vegetable soybean
Source: PLoS One. 2024 Nov 14;19(11):e0313632. doi: 10.1371/journal.pone.0313632 (PMC11563388; doi:10.1371/journal.pone.0313632)
Supplement: Attachment — Submitted filename: Reviewer Comments.pdf [file pone.0313632.s005.pdf]

Reviewer 1

The number of repetitions for the sample in the field experiment is insufficient. In a controlled greenhouse setting, three repetitions may be adequate for a simple test. However, in the field, where numerous uncontrolled factors can influence gene expression, using only three repetitions for each cultivar is not acceptable.

Reviewer 2

I recommend this manuscript for publication in PLOS ONE following minor revisions. The revisions are mostly aimed at interpretation of the results and clarity of the language such that it would be accessible to a larger audience, hence a greater contribution.

• The authors have identified nutritional traits-related DEGs effectively, but such understanding could be even further enriched when a more mechanistic explanation is provided about how DEGs regulate nutrient composition. For example, there is a place for further strengthening the discussion in lines 236-245 by complementing the information with more biological context concerning the functional implication of such genes.

• Similarly, the pathways and genes identified from KEGG enrichment analyses in lines 270-278 could be elaborated to further account for the biological importance of DEGs with respect to nutrient biosynthesis and the stress response.

• Although the manuscript contains lots of data on nutritional composition, no detailed comparison of metabolic pathways between fresh and common soybean cultivars is done in the manuscript. On lines 290-299, to observe which metabolic changes have taken place, more contexts need to be discussed.

• The manuscript does comply with the PLOS ONE data-sharing policy. Lines 126-128 would also read better if the accession numbers or links for RNA-seq were placed directly within the text to make the data more readable to interested readers and researchers who might want to scrutinize it.

• The manuscript is generally well-written, although some places require minor refinement with regard to chosen language in order to enhance clarity. For example, complicated sentences in the introduction should be simplified for easier reading (lines 34-40).

• The manuscript is well-structured and well-organized with the discussion, particularly lines 384-394, showing a need for comparison to related studies on vegetable soybean varieties. More context background would be able to emphasize the relevance and novelty of the study by showing how these findings relate to existing literature.

• The study will interest agricultural researchers and breeders alike, especially with the current upsurge in functional foods. The authors could do a better explanation on practical applications and limitations of this research into breeding programs on lines 431-435 to place more emphasis on the importance of the study in fronting nutritionally improved soybean varieties.
